# The relevant research of adverse childhood experiences and “risky drinking” in children of alcoholics in China

**DOI:** 10.1186/s12888-023-04526-0

**Published:** 2023-01-13

**Authors:** Guangqiang Sun, Tingfang Wu, Chengbing Huang, Mingchao Yu, Yan Guo, Xihua Zhu, Xin Yu, Yujia Qiu

**Affiliations:** 1 The National Clinical Research Center for Mental Disorders & Beijing Key Laboratory of Mental Disorders, Beijing Anding Hospital, Capital Medical University, Beijing, China; 2 Advanced Innovation Center for Human Brain Protection, Capital Medical University, Beijing, China; 3 The Third People’s Hospital of Huai’an, Huai’an, China; 4 Zigong Mental Health Center, Zigong, China; 5 The Third Hospital of Heilongjiang, Bei’an, China; 6 Peking University Sixth Hospital, Peking University Institute of Mental Health, NHC Key Laboratory of Mental Health (Peking University), National Clinical Research Center for Mental Disorders (Peking University Sixth Hospital), Beijing, China

**Keywords:** Adult children of alcoholics, Adverse childhood experience, Risky drinking

## Abstract

**Objective:**

To determine whether adverse childhood experiences (ACEs) of children of alcoholics (COA) in male were associated with their current “risky drinking”.

**Methods:**

This case–control study used the Alcohol Use Disorder Identification Test (AUDIT, cutoff is 7) to divide the participants into two groups, a “risky drinking” group (N = 53) and a "non-risky drinking” group (N = 97). Demographic data, Adverse Childhood Experiences-International Questionnaire (ACE-IQ), the Hamilton Anxiety Rating Scale (HAMA), the Hamilton Depression Rating Scale (HAMD) and the Mini-International Neuropsychiatric Interview (MINI) were used for assessment. The specific relationships between ACEs and “risky drinking” were explored.

**Results:**

Respondents ranged in age from 29.70 ± 6.72 years; 74.5% were females; 94.7% were of Han nationality; 56.7% had a level of education above high school; 12% had no formal or stable job. There was difference in attitude to self-drinking between two groups (*P* < 0.001). The “risky drinking” group was more likely to have experienced a major depressive episode (*P* < 0.05), nonalcohol psychoactive substance use disorder (*P* < 0.01) and bulimia nervosa (*P* < 0.05), and they also experienced more physical abuse (*P* < 0.05), community violence (*P* < 0.001) and collective violence (*P* < 0.01). In a single factor logistic regression, physical abuse, community violence and collective violence were associated with a two to 11- fold increase in “risky drinking” in the adult COA, and in multiple factor logistic regression, community violence showed a graded relationship with “risky drinking”.

**Conclusion:**

The childhood adverse experiences contribute to “risky drinking” in COA. This finding in the Chinese context have significant implications for prevention not only in China but in other cultures. There must be greater awareness of the role of ACEs in the perpetuation of alcoholism.

## Introduction

Alcohol dependence was marked by compulsive drinking, withdrawal symptoms and increased tolerance to alcohol [1]. The prevalence of this mental disorder in Europe and the United States was about 3.5–13.5% [2] and 3–3.8% in China [3]. Thus, there were 7.8 million children, were affected by parents who suffered alcohol dependence living with them in the U.S. [4]. There lacked data of children of parents with alcohol dependence (COPAD) in China, but according to the prevalence of alcohol use disorder and the national population [3, 5], the number would not be less than that in western countries.

Although the number of offspring with alcoholic parents had not been counted worldwide, limited research had shown that alcohol dependence had an intergenerational impact with psychological and social implications. The offspring of those with alcoholism were at risk of experiencing the negative effects of parental alcohol dependence [6]. Compared with the children of those without alcoholism, these children had a higher vulnerability to developing “risky drinking” [7] and other negative social and mental outcomes, including mood problems, suicide, school dropout, marital discord, and work and social relationship problems [7–9]. “Risky drinking”, which was defined as consuming ≥ 5 standard drinks on a single occasion at least monthly [10], might lead to later heavy drinking and had been associated with a 60% increased risk of developing alcoholism, in which complicated genetic factors may play a role [11, 12]. Higher rates of alcoholism had been found in the offspring of an alcoholic twin than in the children of the nonalcoholic twin [13], indicating that when genetic factors were excluded the development of alcoholism in COA might mainly be due to environmental factors. In a Vietnamese twin study, the baseline rates of alcohol use problems were higher in children of alcoholic parents than in those of the nonalcoholic parents [13]. A Swedish retrospective cohort study showed that children of parents with alcohol use disorders had increased alcohol use problems [14]. Other studies found that parental alcohol dependence impaired family functioning and increased the risk of violence and physical abuse, criminal behavior and parental separation [15]. Given that the children of alcoholics who engaged in risky drinking were the most susceptible group to developing more serious mental health problems, including alcohol dependence, it was necessary to explore how the known adverse childhood experiences impacted later mental health problems [6].

ACEs included emotional abuse, physical abuse, sexual abuse, emotional neglect, physical neglect, and family dysfunction (domestic violence, parental drug use, divorce and incarceration) [16]. In a meta-analysis of Chinese children, 27% suffered physical abuse, 20% suffered emotional abuse, 9% suffered sexual abuse, and 26% suffered neglect [16, 17]. and higher ACEs scores were associated with worse psychological functioning, such as anxiety and depression, and externalizing symptoms, such as impulsive and aggressive behavior [18]. Limited research focused on Chinese populations had shown that greater ACEs exposure was associated with alcohol abuse [16, 19]. Meanwhile, among Chinese general medical students, a higher prevalence of “risky drinking” existed among people with parental alcoholism [20]. Students who had experienced an ACEs had a two to four fold increase in “risky drinking” compared to those who experienced no ACE [20]. Studies had indicated that COA had a higher risk of risky drinking, but the necessity of comparing the environmental factors still existed when analyzing the risk factors for alcohol use problems among COA.

Existing studies examining COA came mostly from Western countries, which had quite a different cultural background than Asian countries [21]. It was important that sources of bias and confounders, such as country, ethnicity, cultural background, were strictly considered and controlled. Meanwhile, we needed to exclude maternal drinking from the present study to decrease a confounding variable—parental gender—as the incidence of alcohol dependence was significantly higher in men than women [2]. In addition, other studies found that female alcoholism had less alcohol consumption and were less likely to have behavioral problems associated with heavy drinking [22]. Women tended to develop alcohol dependence at a later age [22]. Therefore, the effect of maternal drinking on children was relatively small. To our knowledge, this is the first study in China focusing on COA seeking an association with ACEs. Thus, we can better understand the relationship between adverse childhood experiences and current risky drinking among COA in male in Chinese culture. The study had implications for other Asian population. This study hypothesized that the “risky drinking” group would have suffered more ACEs than the comparison group.

## Materials and methods

### Participants

In this case–control study, participants were enrolled via an advertisement and screened from August 2020 to February 2021 at Peking University Sixth Hospital.

The inclusion criteria for those in the risky drinking group included the following:

- biological father could be diagnosed as alcohol dependence based on ICD-10 (International Classification of Diseases, tenth revision) criteria;
- participant was aged 18 to 45;
- participant had an AUDIT score ≥ 7 (This indicates that participants were at higher risk for alcohol dependence).

The exclusion criteria for those in the risky drinking group included the following:

- severe physical or neurological disease;
- history of loss of consciousness or learning disability;
- mother drank during pregnancy or could be diagnosed with maternal alcohol use disorder based on ICD-10 criteria;
- either parent was diagnosed with schizophrenia, bipolar disorder or dementia.

The inclusion criteria for those in the non-risky drinking group differed from the risky group was that AUDIT score < 7. The exclusion criteria for those in the non-risky drinking group were the same as for the risky drinking group.

A total of 213 participants were enrolled after the first screening, A total of 161 persons provided informed consent in writing, and the remaining persons did via WeChat. Eighty-four participants were not in Beijing and were interviewed via video phone, and 77 persons came to Peking University Sixth Hospital and were interviewed face to face. The protocol took one hour to complete. Finally, 150 participants met all inclusion criteria and did not meet the exclusion criteria.

### Study design

The two-dimensional code of the advertisement was sent to discharged inpatients, follow-up groups, or posted in the outpatient department. There was a three-phase screening process. One attending psychiatrist, trained to perform the evaluation, interviewed the participants and completed all questionnaires. Simple feedback was given to the participants.

Six questionnaires were used in the interview.

- Demographic form recorded data including sex, age, race, education, occupation, income, marriage, attitudes toward their father's drinking, attitudes toward self-drinking and knowledge of self-help groups (see Table 1).
- The Alcohol Use Disorder Identification Test (AUDIT) was used to divide the participants into the “risky drinking group” and “non-risky drinking group” using a cutoff of 7, which had been tested in China and found to be the best score to identify “risky drinking” in the Chinese population. Cronbach’s alpha was 0.782, and the item-level content validity index was 0.83 [23].
- A Chinese version of the ACE adapted from World Health Organization (WHO) ACE-IQ [16], was used and included 29 items and 13 classifications (emotional neglect; physical neglect; emotional abuse; physical abuse; sexual abuse; alcohol and/or drug abuser in the household; living with someone chronically depressed, mentally ill, institutionalized or suicidal; living with incarcerated household member; one or no parents; parental separation or divorce; family violence; bullying; community violence; and collective violence). The participants were asked to choose the frequency of the ACE items, from “never” to “many times” to account for the level of exposure. Regarding internal consistency, Cronbach’s alpha was 0.83, and all the subscales showed good test–retest reliability, with intraclass correlations ranging between 0.78 and 0.90 [16].
- The Hamilton Anxiety Rating Scale (HAMA) was used to rate anxiety with a cutoff score of 14 to divide the different classes. The validity index for the total score was 0.93, and the validity correlation index of the subscales was 0.83–1 (*P* < 0.01). The reliability index was 0.36 [24].
- The 17-item Hamilton Depression Rating Scale (HAMD-17) was used to rate the degree of depression with a cutoff score of 17 used to divide the participants into depressed and nondepressed groups. The validity index of the total score was 0.99, and the validity correlation index of the subscales was 0.78–0.98 (*P* < 0.01). The reliability index was 0.92 [25].
- The Mini-International Neuropsychiatric Interview (MINI) was used to screen for mental disorders [26]. The concurrent validity within interviewers and a retest were 0.94 (*P* < 0.01) and 0.97 (*P* < 0.01)respectively, and the criterion validity was between 0.764 and 0.880 [27].

**Table 1:** Adverse childhood experience between “risky drinking” and “non-risky” drinking groups among adult children of alcoholics

	Risky drinking group *N* = 53	Non-risky drinking group *N* = 97	χ^2^/ Z	*P*
Sex***				
Male	24(45.3%)	14(14.4%)	17.244	< 0.001
Female	29(54.7%)	83(85.6%)		
Age	30.79 ± 5.627	28.9 ± 6.015	-1.901	.057
Race				
Han	49(92.5%)	93(95.9%)	.769	.454
Others	4(7.5%)	4(4.1%)		
Education			1.234	.523
Middle school/ Technical school	2(3.8%)	8(8.2%)		
College and University	32(60.4%)	53(54.6%)		
Master and above	19(35.8%)	36(37.1%)		
Occupation			.743	.435
Formal job	45(84.9%)	87(89.7%)		
No formal job	8(15.1%)	10(10.3%)		
Income (RMB)			4.230	.057
< 5000	17(32.1%)	48(49.5%)		
≥ 5000	36(67.9%)	49(50.5%)		
Marriage			2.422	.546
Single or divorced	36(68.0%)	64(66.0%)		
Married	17(32.1%)	33(34.0%)		
Attitude to father’s drinking			5.682	.061
Accept	17(32.1%)	15(15.5%)		
Object	32(60.4%)	74(76.3%)		
Neutral	4(7.5%)	8(8.2%)		
Attitude to self-drinking***			24.382	< 0.001
Enjoy	20(37.7%)	6(6.2%)		
Neutral	25(42.7%)	62(63.9%)		
Hate	8(15.1%)	29(29.9%)		
Feel shame of father’s drinking			3.797	.159
Yes	12(22.6%)	37(38.1%)		
No	23(43.4%)	35(36.1%)		
Neutral	18(34.0%)	25(25.8%)		
Knowledge about AA Group			.085	.865
Nearly nothing	27(50.9%)	47(48.5%)		
Know it	26(49.1%)	50(51.5%)		
Knowledge about Al-Anon Group			.383	.565
Nearly nothing	47(88.7%)	89(91.8%)		
Know it	6(11.3%)	8(8.2%)		
*Adverse Childhood Experience*				
Emotional neglect	13(24.5%)	29(29.9%)	.490	.570
Physical neglect	38(71.7%)	57(58.8%)	2.469	.156
Emotional abuse	45(84.9%)	77(79.4%)	.689	.510
Physical abuse*	40(75.5%)	53(54.6%)	6.313	< 0.05
Sexual abuse	21(39.6%)	43(44.3%)	.310	.608
Household member treated violently	49(92.5%)	91(93.8%)	.102	.743
Community violence***	50(94.3%)	58(59.8%)	20.289	< 0.001
Collective violence**	10(18.9%)	4(4.1%)	7.149	< 0.01
Incarcerated household member	4(7.5%)	8(8.2%)	.000	1.000
Bullying	32(60.4%)	42(43.3%)	3.999	.060
Someone chronically depressed, mentally ill, institutionalized or suicidal	12(22.6%)	21(21.6%)	.020	.889
Parental separation	20(37.7%)	32(33.0%)	.341	.559
No parent	6(11.3%)	10(10.3%)	.037	.848
Patterns of ACEs (%)			26.649	.063
0	0(0%)	0(0%)		
1	0(0%)	3(3.1%)		
2	0(0%)	1(1.0%)		
3	1(1.9%)	2(2.1%)		
4 and above	96(98.1%)	47(93.8%)		
*Comorbidities*				
*HAMA*			*.832*	*.425*
< 14	38(71.7%)	76(78.4%)		
≥ 14	15(28.3%)	21(21.6%)		
HAMD-17*			*5.867*	< 0.05
< 17	33(62.3%)	78(80.4%)		
≥ 17	20(37.7%)	19(19.6%)		
*MINI*				
Current Major Depressive Episode*	20(37.7%)	21(21.6%)	4.465	< 0.05
Past Major Depressive Episode	34(64.2%)	48(49.5%)	2.975	.090
Dysthymia	15(28.3%)	17(17.5%)	2.372	.146
Current suicide idea or attempt	11(20.8%)	12(12.4%)	1.856	.235
Past suicidal attempt	15(28.3%)	19(19.6%)	1.485	.308
Current (Hypo)Manic Episode	0(0%)	1(1.0%)	.550	1.000
Past (Hypo)Manic Episode	10(18.9%)	7(7.2%)	4.630	.056
Current Panic Disorder	4(7.5%)	6(6.2%)	.102	.743
Past Panic Disorder	12(22.6%)	12(12.4%)	2.690	.109
Agoraphobia	8(15.1%)	7(7.2%)	2.363	.156
Current Social Phobia	10(18.9%)	11(11.3%)	1.613	.225
Current Obsessive–compulsive Disorder	7(13.2%)	7(7.2%)	1.454	.250
Past PTSD	4(7.5%)	5(5.2%)	.348	.721
Non-alcohol Psychoactive Substance Use Disorder**	10(18.9%)	4(4.1%)	8.805	< 0.01
Psychotic disorder	6(11.3%)	6(6.2%)	1.228	.347
Anorexia Nervosa	5(9.4%)	5(5.2%)	1.009	.495
Bulimia Nervosa*	11(20.8%)	6(6.2%)	7.239	< 0.05
Generalized Anxiety Disorder	15(28.3%)	24(24.7%)	.226	.698
Antisocial Personality Disorder	2(3.8%)	1(1.0%)	1.315?	.285

*AA* Alcoholics Anonymous; *p < 0.05; ** *p* < 0.01; *** *p* < 0.001

The study was approved by the Institutional Review Board of Peking University Sixth Hospital (No. 202046). The Clinical Report Form contained an introduction to the study and informed consent, which stated that the participants joined the study voluntarily. Confidentially was assured. The participants who were not in Beijing verbally agreed to the informed consent by WeChat. No payment was given to the participants for joining this study. All the authors gave their consent for publication.

### Statistical analysis

SPSS 26.0 was used to perform the statistical analysis. We compared the variables of demographic data, ACE-IQ, HAMA, HAMD-17, MINI, AUDIT between two groups. Nonparametric tests were used to compare the only continuous variable of age. Chi-square analyses and Z analysis were used to compare the remaining variables. The simple factor binary logistic regression compared risky drinking (AUDIT ≥ 7) as a dependent variable and adverse childhood experience and other variables that showed significant differences between the two groups as independent variables. In a multifactor binary logistic regression, we took all independent variables as covariates. In both regression analyses, we calculated the adjusted odds ratios (OR) and 95% confidence intervals (95% CI) for the associations between risky drinking and adverse childhood experiences. In all analyses, *P*-values below 0.05 were considered statistically significant.

## Results

### Study participant characteristics

The mean age of the 150 participants in the total study was 29.70 with an SD of ± 6.722 years (range, 18 to 45 years). A total of 74.5% were females (*n* = 112), and 94.7% were of Han nationality (*n* = 142). Seven percent graduated from middle school (*n* = 11) or technical school, and 56.7% graduated or were currently in college or university (*n* = 85). There were 12% with no formal education and stable job (*n* = 18), and 43.3% earned less than 5000 RMB per month (*n* = 65). A total of 66.7% were single or divorced (*n* = 100) (see Table 1). The diagnosis of 140 (93.3%) of the participants' fathers was made by the interviewer based on the history that the offspring provided, and only 10 fathers were diagnosed by psychiatric hospitals.

### Features compared between two groups

The “risky drinking” group had fewer females than the “non-risky drinking” group (54.7% vs. 85.6%, χ^2^ = 17.244, *P* < 0.05). There was statistical difference in attitude to self-drinking between two groups (χ^2^ = 24.382, *P* < 0.001). The “risky drinking” group was more likely to enjoy drinking, while the “non-risky drinking” group was more likely to hate drinking. More “risky-drinking” people had suffered a current major depressive episode (37.7% vs. 21.6%, Z = 4.465, *P* < 0.05), nonalcohol psychoactive substance use disorder (18.9% vs. 4.1%, Z = 8.805, *P* < 0.01), and bulimia nervosa (20.8% vs. 6.2%, Z = 7.239, *P* < 0.05). More people in the “risky drinking” group experienced physical abuse (75.5% vs. 54.6%, Z = 6.313, *P* < 0.05), community violence (94.3% vs. 59.8%, Z = 20.289, *P* < 0.001 = and collective violence (18.9% vs. 4.1%, Z = 7.149, *P* < 0.01). All other ACEs and mental disorders screened by MINI showed no significant differences. Comparing the number of ACEs showed that both groups suffered more than one kind of ACE, with most of them having suffered 4 or more ACEs; the risky drinking group suffered more ACEs, but the difference was not statistically significant (see Table 1).

Comparing the frequency of items on the ACE, more COA in the “risky drinking” group suffered physical abuse regardless of the frequency (χ^2^ = 8.659, χ^2^ = 7.934, *P* < 0.05), and more COA in the same group suffered community violence with frequencies of “a few times” and “many times” (χ^2^ = 17.310, *P* = 0.001). The item “Did you experience the deliberate destruction of your home due to any of these events?” showed a difference between the two groups (χ^2^ = 7.521, *P* < 0.05), because a few people in the risky drinking group suffered the experience one or more times, but no participant in the non-risky drinking group had this kind of experience. There was no significant difference between the two groups in emotional neglect, physical neglect, domestic violence, emotional abuse, sexual abuse or bullying (see Table 2).

**Table 2:** Specific ACE items compared between different groups

Items^a^	Frequency^b^	Risky Drinking Group	Non-risky Drinking Group	χ^2^/ Z	*P*
*Emotional Neglect*					
1.1 Did your parents/guardians understand your problems and worries?				6.195	0.185
	never-0	7(13.2%)	22(22.7%)		
	Rarely-1	19(35.80%)	22(22.70%)		
	Sometimes-2	16(30.20%)	28(28.90%)		
	Most of the time-3	9(17%)	14(14.40%)		
	Always-4	2(3.80%)	11(11.30%)		
1.2 Did your parents/guardians really know what you were doing with your free time when you were not at school or work?				0.687	0.953
	never-0	10(18.90%)	15(15.50%)		
	Rarely-1	15(28.30%)	25(25.80%)		
	Sometimes-2	12(22.60%)	26(26.80%)		
	Most of the time-3	8(15.10%)	14(14.40%)		
	Always-4	8(15.10%)	17(17.50%)		
*Physical Neglect*					
2.1 How often did your parents/guardians not give you enough food even when they could easily have done so?				3.416	0.332
	Never-0	48(90.60%)	93(95.90%)		
	Once-1	1(1.90%)	1(1.00%)		
	A few times-2	4(7.50%)	2(2.10%)		
	Many times-3	0	1(1%)		
2.2 Were your parents/guardians too drunk or intoxicated by drugs to take care of you?				3.496	0.350
	Never-0	16(30.20%)	40(41.20%)		
	Once-1	1(1.90%)	4(4.10%)		
	A few times-2	13(24.50%)	17(17.50%)		
	Many times-3	0	1(1%)		
2.3 How often did your parents /guardians not send you to school even when it was available?				0.557	0.757
	Never-0	52(98.10%)	94(96.90%)		
	Once-1	1(1.90%)	2(2.10%)		
	A few times-2	0.00%	2(1.00%)		
	Many times-3	0	0		
*Domestic Violence*					
3.6 Did you see or hear a parent or household member in your home being yelled at, screamed at, sworn at, insulted or humiliated?				2.784	0.595
	Never-0	6(11.30%)	8(8.20%)		
	Once-1	0	2(2.10%)		
	A few times-2	13(24.50%)	25(25.80%)		
	Many times-3	32(60.40%)	61(62.90%)		
3.7 Did you see or hear a parent or household member in your home being slapped, kicked?				2.149	0.708
	Never-0	18(34.00%)	38.10%		
	Once-1	6(11.30%)	9(9.30%)		
	A few times-2	19(35.80%)	34(35.10%)		
	Many times-3	9(17%)	18(19.40%)		
3.8 Did you see or hear a parent or household member in your home being hit or cut with an object, such as a stick (or cane), bottle, club, knife, whip etc.?				6.319	0.097
	Never-0	34(64.20%)	73(75.30%)		
	Once-1	6(11.30%)	2(2.10%)		
	A few times-2	8(15.10%)	12(12.40%)		
	Many times-3	5(9.40%)	10(10.30%)		
*Emotional Abuse*					
4.1 Did a parent, guardian or other household member yell, scream or swear at you, insult or humiliate you?				3.658	0.301
	Never-0	8(15.10%)	23(23.70%)		
	Once-1	1(1.90%)	6(6.20%)		
	A few times-2	23(43.40%)	32(33.00%)		
	Many times-3	21(39.60%)	36(37.10%)		
4.2 Did a parent, guardian or other household member threaten to, or actually, abandon you or throw you out of the house?				3.384	0.336
	Never-0	33(62.30%)	69(71.10%)		
	Once-1	3(5.70%)	6(6.20%)		
	A few times-2	12(22.60%)	11(11.30%)		
	Many times-3	5(9.40%)	11(11.30%)		
*Physical Abuse*					
4.3 Did a parent, guardian or other household member spank, slap, kick, punch or beat you up?				8.659	< 0.05
	Never-0	13(24.50%)	44(45.40%)		
	Once-1	8(15.10%)	10(10.30%)		
	A few times-2	17(32.10%)	30(30.90%)		
	Many times-3	15(28.30%)	13(13.40%)		
4.4 Did a parent, guardian or other household member hit or cut you with an object, such as a stick (or cane), bottle, club, knife, whip, etc.?				7.934	< 0.05
	Never-0	41(77.40%)	77(79.40%)		
	Once-1	0	7(7.20%)		
	A few times-2	7(13.20%)	11(11.30%)		
	Many times-3	5(9.40%)	2(2.10%)		
*Sexual Abuse*					
4.5 Did someone touch or fondle you in a sexual way when you did not want them to?				3.068	0.547
	Never-0	41(77.40%)	69(71.10%)		
	Once-1	5(9.40%)	11(11.30%)		
	A few times-2	5(9.40%)	15(15.50%)		
	Many times-3	2(3.80%)	1(1.00%)		
4.6 Did someone make you touch their body in a body in a sexual way when you did not want them to?				2.990	0.560
	Never-0	47(88.70%)	90(92.80%)		
	Once-1	3(5.70%)	4(4.10%)		
	A few times-2	2(3.80%)	2(2.10%)		
	Many times-3	1(1%)	1(0.70%)		
4.7 Did someone attempt oral, anal, or vaginal intercourse with you when you did not want them to?				0.928	0.819
	Never-0	51(96.20%)	92(94.80%)		
	Once-1	1(1.90%)	3(3.10%)		
	A few times-2	1(1.90%)	1(1.00%)		
	Many times-3	0	1(1%)		
4.8 Did someone actually have oral, anal, or vaginal intercourse with you when you did not want them to?				1.286	0.732
	Never-0	52(98.10%)	94(96.90%)		
	Once-1	0.00%	1(1.00%)		
	A few times-2	0.00%	1(1.00%)		
	Refused	1(1.90%)	1(1%)		
*Bullying*					
5.1 How often were you bullied?				4.280	0.369
	Never-0	21(39.60%)	55(56.70%)		
	Once-1	8(15.10%)	9(9.30%)		
	A few times-2	18(34%)	24(24.70%)		
	Many times-3	5(9.40%)	8(8.20%)		
*Community Violence*					
6.1 Did you see or hear someone being beaten up in real life?				20.492	—
	Never-0	6(11.30%)	42(43.30%)		
	Once-1	5(9.40%)	12(12.40%)		
	A few times-2	29(54.70%)	35(36.10%)		
	Many times-3	13(24.50%)	8(8.20%)		
6.2 Did you see or hear someone being stabbed or shot in real life?				17.310	0.001
	Never-0	30(56.60%)	80(82.50%)		
	Once-1	6(11.30%)	10(10.30%)		
	A few times-2	15(28.30%)	5(5.20%)		
	Many times-3	2(3.80%)	2(2.10%)		
6.3 Did you see or hear someone being threatened with a knife or gun in real life?				18.609	—
	Never-0	27(50.90%)	78(80.40%)		
	Once-1	5(9.40%)	9(9.30%)		
	A few times-2	18(34%)	9(9.30%)		
	Many times-3	3(5.70%)	1(1%)		
*Collective Violence*					
7.1 Were you forced to go and live in another place due to any of these events?				4.402	0.111
	Never-0	50(94.30%)	93(95.90%)		
	Once-1	0	3(3.10%)		
	A few times-2	3(5.70%)	1(1%)		
	Many times-3	0	0		
7.2 Did you experience the deliberate destruction of your home due to any of these events?				7.521	< 0.05
	Never-0	49(92.50%)	97(100%)		
	Once-1	3(5.70%)	0		
	A few times-2	1(1.80%)	0		
	Many times-3	0	0		
7.3 Were you beaten up by soldiers, police, militia, or gangs?				5.603	0.061
	Never-0	50(94.30%)	97(100%)		
	Once-1	2(3.80%)	0		
	A few times-2	1(1.90%)	0		
	Many times-3	0	0		
7.4 Was a family member or friend killed or beaten up by soldiers, police, militia, or gangs?				7.521	0.057
	Never-0	49(92.50%)	97(100%)		
	Once-1	2(3.80%)	0		
	A few times-2	1(1.90%)	0		
	Many times-3	1(1.90%)	0		

^a^The item which need to be counted by dichotomy method has been excluded from this table

^b^The frequency does not show when both of the groups are zero

### Association of risky drinking and adverse childhood experiences

We chose the variables that showed a significant difference between the two groups to perform a single factor logistic regression, which were HAMD scores ≥ 14 (OR = 2.488, *P* < 0.05), current major depressive episode (OR = 2.193, *P* < 0.05), nonalcohol psychoactive substance use disorder (OR = 5.407, *P* < 0.01), bulimia nervosa (OR = 3.972, *P* < 0.05), physical abuse (OR = 2.554, *P* < 0.05), community violence (OR = 11.207, *P* < 0.01) and collective violence (OR = 5.407, *P* < 0.01). Adverse childhood experiences showed a graded relationship with “risky drinking” in adult COA. Depression, nonalcohol psychoactive substance use and bulimia nervosa increased the risk of “risky drinking” two to five fold, while physical abuse, community violence and collective violence increased the risk of “risky drinking” two to 11- fold in adults with COA (see Table 3).

**Table 3:** Single factor logistic regression: relationship of potential factors and risky drinking

	OR	95% CI		*P*
Sex (male)	.204	.093	.446	< 0.001
Attitude to self-drinking	.288	.156	.533	< 0.001
HAMD (≥ 17)	2.488	1.177	5.258	< 0.05
MINI				
Current Major Depressive Episode	2.193	1.050	4.580	< 0.05
Non-alcohol Psychoactive Substance Use Disorder	5.407	1.605	18.215	< 0.05
Bulimia Nervosa	3.972	1.376	11.463	< 0.05
Adverse Childhood Experience				
Physical Abuse	2.554	1.216	5.367	< 0.05
Community violence	11.207	3.264	38.484	< 0.001
Collective violence	5.407	1.605	18.215	< 0.01

After controlling for the covariates of sex, attitude toward self-drinking, HAMD score, current depression, nonalcoholic psychoactive substance use and bulimia nervosa, which also impacted “risky drinking”, people who experienced community violence were 14 times more likely to have a “risky drinking” problem (see Table 4).

**Table 4:** Multifactor logistic regression of relationship between ACE and risky drinking

	OR	95%CI		*P*
Sex (male)	.106	.035	.325	
Attitude to self-drinking	.156	.066	.370	< 0.001
HAMD (≥ 17)	3.317	1.118	9.835	.031
MINI				
Current Major Depressive Episode	.961	.245	3.766	.954
Non-alcohol Psychoactive Substance Use Disorder	2.608	.521	13.056	.243
Bulimia Nervosa	8.189	1.824	36.766	< 0.01
Adverse Childhood Experience				
Physical Abuse	1.973	.667	5.835	.219
Community violence	14.372	3.318	62.245	< 0.001
Collective violence	5.171	.898	29.774	.066

## Discussion

The major finding of this study was that the entire sample suffered at least one ACE, and that the COA with “risky drinking” had a broader range of patterns of adverse childhood experiences and a higher frequency than the non-risky drinking group, including physical abuse, community violence and collective violence. These factors had a graded relationship with “risky drinking” in the COA. Physical abuse, community violence and collective violence showed a two to 11- fold increase in “risky drinking” in the adult COA before controlling for the covariate of comorbidities. Community violence was still associated with a 14- fold increase after controlling for the covariates. In addition, we found that “risky drinking” group enjoyed self-drinking more, “non-risky drinking” group hated self-drinking more.

In this study, we found that all of the COA reported at least one ACE, but there was no significant difference in the number of ACEs between two groups, which could indicate that there was a strong association between alcohol-abusing parents and ACEs in children. One of the reason was that living with intoxicated parents probably led to children facing dysfunctional parenting, emotional and physical neglect and/or abuse, which will affect parent-children bonding and their feeling of safety [21, 28–30]. In addition, alcohol-using parents appeared to pass on drinking patterns through being a negative role model for responding to life difficulties or conflicts [31, 32]. Therefore, COA might learn maladaptive responses in their school and social relationships, witch was more strongly related to the content nature of ACEs, rather than the number of ACEs. Children might also pursue self-medication to release their negative emotions, such as fear, shame, phobia, anxiety and/or depression. Self-medication included the use of psychoactive substances and other risky behaviors [33]. Additionally, as they lived in unsafe surroundings, more physical abuse or community and collective violence would be experienced or witnessed by the COA [31, 32].

It was shown in this study that in the “risky drinking” group, community violence, domestic violence, emotional abuse, and physical abuse were the top four adverse experiences, and in the “non-risky drinking group”, domestic violence, emotional abuse, community violence and physical neglect had the highest prevalence, which was similar to findings in Hong Kong [16]; however, these data contrasted with the results from the US and Canada where there appeared to be higher exposure to household dysfunction (including neglect and abuse) rather than violence [34–36]. The high level of domestic violence toward both family members and COA reflected a feature of a different culture, such as rigid gender roles, endorsement of physical punishment and absolute parental authority [16, 37–39]. Community violence showed differences, and this factor might be added to the other ACEs that already influenced the health of COA. Community violence reflected a harsher living environment, and this finding was in accordance with the results of another study that showed the positive relationship between a disadvantaged community and risky drinking [40]. Part of the condition described as community violence and collective violence, such as “being threatened with a gun in real life, deliberate destruction of your home or having been beaten up by soldiers, police, militia, or gangs”, was not frequent in Chinese society; therefore, the understanding of community violence was different in Western and Eastern countries. The finding that the rest of the ACEs, excluding community violence, showed no significant impact on “risky drinking” might be due to the high rate of ACEs in both groups, and COA who showed “risky drinking” experienced more adverse childhood experiences than the general population.

In this experiment, we found that there were significant differences in attitude to self-drinking between the two groups, in addition to the significant differences in ACEs, Other studies had shown that ACEs could affect the acquired changes in brain structure and function, personality development, and interpersonal relationships of individuals [41]. In a study on adolescent drinking attitudes, they found that lack of parental presence was a risk factor for alcohol consumption among adolescents [42]. The possible reason was that adolescents were closely connected with their parents. According to social learning theory, adolescents were willing to learn behaviors from those around them [43]. Therefore, when individuals suffer more ACEs, they prefered to deal with the problem through drinking, leading to difference in attitude to self-drinking between two groups.

Regarding the comorbidities, the “risky drinking” group was more likely to suffer current depressive episodes, non-alcohol psychoactive substance use disorder and bulimia nervosa than the “non-risky drinking” group. It was difficult to determine the chronological sequence of “risky drinking” and other mental health problems because of the cross-sectional design of this study. This result was in accordance with a meta-analysis, which showed that externalizing problems in some studies and depression tended to be positively associated with alcohol use, but there was no clear association between alcohol problems and anxiety [44]. A potential explanation was that people who had a tendency toward behavioral disinhibition were more likely to be involved in restricted actions, especially those who had experienced adverse and high-risk living environments [45–47]. Another mechanism was related to the presence of an internalizing pathway, which was also known as “self-medication” or “tension reduction” [48], as previously elaborated. COA often lacked adaptive social skills when facing difficulties; consequently, they were more likely to have mental health problems, including “risky drinking” and depression [30, 49]. In a study conducted by our team in 2011, it was shown that most alcoholics who had a comorbidity of social anxiety declared that they had social anxiety before drinking and that drinking decreased the anxiety symptoms [50]. Comorbidities could interact with risky drinking or be a negative outcome of ACEs. In cross-sectional studies, comorbidities should be controlled as covariates in the logistic regression model.

In this study, through self-selection, most of the respondents were young females who were highly educated, had a stable job and had a relatively high income. This seemed counterintuitive and made the findings even more striking than if the group was marginal regarding their education, employment and income. It might be that females were more likely to be aware of fathers’ drinking problems and had a curiosity to know more about what is wrong with their father. In the “risky drinking” group, there were more males. One possible reason was that sons of alcoholics were more sensitive to the euphoric and stimulatory effects of alcohol [12]. They initiated repeated drinking to avoid negative hedonic effects [12]. Thus, sons of alcoholics were likely to develop alcohol use problems.

This study also had the following deficiencies. Although a structured interviewing tool was used to assess the participants, we gathered the history of the probands mainly through the reports of the COA. The exclusion of maternal alcoholics might have risked the introduction of biases to the findings, but as there was a significant discrepancy between the prevalence of male and female alcohol dependence in China (6.6% vs 0.2%) [51], it might not be a major problem for this study in the context of Eastern culture. There existed selection bias in that the participants in this study generally had a high education and stable employment, which were potentially protective factors, and they had a high percentage of ACEs and comorbidities, which might be risk factors. In the future, studies should expand the study population to be more representative of the general public. The ACE measure was used worldwide, but it was still limited regarding the collection of duration, frequency and onset of adverse experiences. As it was a case–control study, we could make the assumption of a causal relationship between risky drinking in COA and ACEs, but if we wanted to clarify the cause and result, future, larger sample cohorts with strict control of confounds should be developed.

The findings of this study are meant to help clinicians focus more on the family of COA and, for the first time in our country, provide data and a theoretical basis for the needed healthcare, psychological support and societal understanding. Schools and medical professionals need to perform more evaluations of the negative childhood experiences of COA and provide interventions for COA. The implications of this study are particularly strong for Asian cultures where awareness and prevention efforts lag.

## Conclusion

COA engaging in “risky drinking” experienced more childhood adversities, such as physical abuse and environmental violence, and had more comorbidities, such as depression and bulimia nervosa. On the basis of the high prevalence of ACEs among the whole sample of COA, even after controlling for the covariates, one pattern of ACEs still had an apparent association with risky drinking. In China, this study was one of very few studies that have focused on COA who were at high risk of developing drinking and other mental health problems. In this case–control study, we aimed to understand the impact of factors influencing the lives of COA and explored possible targets for intervention and prevention. Raising awareness of the association between alcoholism and ACEs has significant implications not only in China but in other Asian cultures. It is essential for the prevention of the intergenerational “transmission” of a propensity to pursue “risky drinking” and alcoholism.

## Data Availability

The datasets used and analysed during the current study available from the corresponding author on reasonable request.
